# Targeted capture and sequencing of *Orientia tsutsugamushi* genomes from chiggers and humans

**DOI:** 10.1016/j.meegid.2021.104818

**Published:** 2021-07

**Authors:** Ivo Elliott, Neeranuch Thangnimitchok, Mariateresa de Cesare, Piyada Linsuwanon, Daniel H. Paris, Nicholas P.J. Day, Paul N. Newton, Rory Bowden, Elizabeth M. Batty

**Affiliations:** a Lao-Oxford-Mahosot Hospital-Wellcome Trust Research Unit, Microbiology Laboratory, Mahosot Hospital, Vientiane, Laos; b Centre for Tropical Medicine and Global Health, Nuffield Department of Medicine, University of Oxford, Oxford, United Kingdom; c Wellcome Centre for Human Genetics, University of Oxford, Oxford, United Kingdom; d Department of Entomology, Armed Forces Research Institute of Medical Sciences, Bangkok, Thailand; e Department of Medicine, Swiss Tropical and Public Health Institute, Basel, Switzerland; f Department of Clinical Research, University of Basel, Basel, Switzerland; g Mahidol-Oxford Tropical Medicine Research Unit, Faculty of Tropical Medicine, Mahidol University, Bangkok, Thailand; h Division of Advanced Technology and Biology, Walter and Eliza Hall Institute of Medical Research, Melbourne, Australia

## Abstract

Scrub typhus is a febrile disease caused by *Orientia tsutsugamushi*, transmitted by larval stage Trombiculid mites (chiggers), whose primary hosts are small mammals. The phylogenomics of *O. tsutsugamushi* in chiggers, small mammals and humans remains poorly understood. To combat the limitations imposed by the low relative quantities of pathogen DNA in typical *O. tsutsugamushi* clinical and ecological samples, along with the technical, safety and cost limitations of cell culture, a novel probe-based target enrichment sequencing protocol was developed. The method was designed to capture variation among conserved genes and facilitate phylogenomic analysis at the scale of population samples. A whole-genome amplification step was incorporated to enhance the efficiency of sequencing by reducing duplication rates. This resulted in on-target capture rates of up to 93% for a diverse set of human, chigger, and rodent samples, with the greatest success rate in samples with real-time PCR C_t_ values below 35. Analysis of the best-performing samples revealed phylogeographic clustering at local, provincial and international scales. Applying the methodology to a comprehensive set of samples could yield a more complete understanding of the ecology, genomic evolution and population structure of *O. tsutsugamushi* and other similarly challenging organisms, with potential benefits in the development of diagnostic tests and vaccines.

## Highlights

• Developed novel probe-based sequence capture method for *Orientia tsutsugamushi* DNA
• Unbiased culture-free sequencing of *O. tsutsugamushi* DNA from chiggers & humans
• Sequence data reveals phylogeographic clustering at different scales.

## 1 Introduction

Scrub typhus is a vector-borne zoonotic disease risking life-threatening febrile infection in humans. The disease is caused by an obligate intracellular Gram-negative bacterium, *Orientia tsutsugamushi.* Scrub typhus has an expanding known distribution, with most disease occurring across South and East Asia and parts of the Pacific Rim.

The genus *Orientia* is classified in the family Rickettsiaceae, a member of the order Rickettsiales. Two species of *Orientia* are currently recognised - *O. tsutsugamushi* and *O. chuto*, the latter known solely from a patient infected in the United Arab Emirates (Izzard et al., 2010). Recent molecular identification of *O. tsutsugamushi* in humans in Chile (Weitzel et al., 2016) and 16S sequences related to *O. tsutsugamushi* in dogs in South Africa (Kolo et al., 2016) and small mammals in Senegal and France (Cosson et al., 2015), and to *O. chuto* in chiggers in Kenya (Masakhwe et al., 2018), suggest the possibility of further species and future taxonomic re-evaluation.

Larval trombiculid mites (chiggers) transmit *Orientia* to vertebrates, including man. The ecology of the disease and the interaction of *Orientia* between vectors, small mammals and humans are complex and relatively poorly understood (Elliott et al., 2019).

A high degree of phenotypic and genotypic diversity has been reported in *O. tsutsugamushi*, in the form of several antigenic types, one dominant, that appear to be widely present throughout Southeast Asia (Kelly et al., 2009). There is also considerable genetic variation, notably in outer membrane proteins such as the 56 kDa and 47 kDa antigens or GroEL. Analysis of 56 kDa sequences from across South and East Asia identified at least 17 clusters of genotypes belonging to 5 identifiable groups (Kim et al., 2017). Multi-locus sequence typing (MLST) (Arai et al., 2013; Duong et al., 2013; Phetsouvanh et al., 2015; Jiang et al., 2013; Sonthayanon et al., 2010) of human isolates from 3 regions of Laos and an isolate from nearby Udon Thani in Northeast Thailand, revealed low levels of population differentiation between geographically close strains with a more distinct, distant population in southern Laos (Phetsouvanh et al., 2015). Recent whole-genome phylogenetic comparisons between 8 strains sequenced with PacBio long-read sequencing revealed relationships that were significantly different from phylogenies created from single-gene or MLST schemes (Giengkam et al., 2015). This illustrates the increased resolution achievable from whole-genome sequencing.

Several factors combine to make genomic studies of *Orientia* infection challenging. The bacterium is an obligate intracellular pathogen, necessitating cell culture for laboratory propagation (Giengkam et al., 2015). *Orientia* is typically collected from a range of specimen types including human whole blood, buffy coat and eschar tissue, rodent blood and organs, and chiggers, and the absolute quantity of *O. tsutsugamushi* DNA present in these specimen types is variable, but frequently low. *Orientia* can only be propagated in cell culture, which is technically demanding (Giengkam et al., 2015), time-consuming, costly, prone to contamination and must be performed in biosafety level 3 facilities (Blacksell et al., 2019). In one study of 155 infected human blood samples tested by 16S PCR, the median pathogen genome load was 0.013 copies/μL, the interquartile range 0–0.334 and the maximum 310 (Sonthayanon et al., 2009), while a recent study from Thailand reported a range of 13.8 to 2252 copies/μL in individual chiggers (Linsuwanon et al., 2018). The *O. tsutsugamushi* genome is relatively poorly defined, with just nine complete genome sequences, and shows a high density of repetitive elements and extreme rates of genomic rearrangement, two added challenges that make innovative approaches to sample preparation, sequencing and analysis essential (Batty et al., 2018; Darby et al., 2007; Nakayama et al., 2010).

In targeted enrichment sequencing, hybridisation of probes to a pool of sequencing libraries is used to make sequencing more efficient. The method is akin to, and works similarly to, whole-exome sequencing where just the “exome” or coding portion of the human genome is sequenced. Targeted enrichment can be useful where the whole genome is not required, or a particular genome of interest is selected from contaminating DNA (Mertes et al., 2011; Summerer, 2009), for example in the metagenomic analysis of multiple virus species, where culture is difficult and costly (Wylie et al., 2015; O'Flaherty et al., 2018; Bonsall et al., 2015), and for *Neisseria meningitidis* directly from cerebrospinal fluid, where culture often fails due to prior antibiotic treatment (Clark et al., 2018). Thus, the method in principle provides an efficient alternative to cell culture combined with whole-genome sequencing for *Orientia.*

In summary, the many difficulties associated with conducting a large-scale study at the whole-genome level of *O. tsutsugamushi* in human, chiggers and small mammal samples prompted the development of a probe-based targeted enrichment sequencing strategy, which was used to examine phylogeographical relatedness of samples collecting in Northern Thailand and elsewhere.

## 2 Results and methods

### 2.1 Design of a comprehensive *O. tsutsugamushi* genomic probe set

Two finished reference strains (Boryong and Ikeda) and seven other available assemblies available (Gilliam: GCF_000964615.1, Karp: GCF_000964585.1, Kato: GCF_000964605.1, TA716: GCF_000964855.1, TA763: GCF_000964825.1, UT144: GCF_000965195.1, UT76: GCF_000964835.1) were used in probe design*.* The complete Boryong strain, used as a reference, was included in the probe design. To cover genes not found in the Boryong genome, or which had high levels of divergence from the Boryong genome, the genome assemblies were reannotated using Prokka v1.11 and predicted open reading frames from all eight genomes were clustered into groups based on ≥ 80% identity at the protein sequence level using Roary v3.6.0 (Page et al., 2015). For each cluster, an alignment of the corresponding DNA sequences (using Clustal Omega (Sievers et al., 2011)) was divided into windows of 120 nt in which every aligned sequence was a candidate probe. Probes were then chosen until every sequence in each cluster was represented by a probe with <10% divergence in DNA sequence, a strategy informed by previous work demonstrating efficient capture with probe target divergence up to 20% (Bonsall et al., 2015) and the requirement to capture as-yet uncharacterised sequences. The reference Boryong gene sequence was always included if it had a representative in the cluster under consideration and sequences that would capture human and rodent genomes (*Rattus norvegicus*) were excluded. The probe design strategy generated a total sequence length of 4.7 Mb which was synthesised as a single Agilent SureSelect probe pool. The FASTA file containing the sequences uploaded for probe design is available at https://doi.org/10.6084/m9.figshare.12546377.

### 2.2 Assembly of a diverse sample collection

Samples containing *O. tsutsugamushi* genetic material were assembled from a variety of sources, including free-living chiggers, wild-living small mammals and clinical samples. A total of 184 small mammals were trapped alive in wire-mesh traps baited with corn, at 5 sites in Northern Thailand: Ban Thoet Thai (20.24°N, 99.64°E), Mae Fahluang district; Ban Song Kwair (20.02°N, 99.75°E) and Ban Mae Khao Tom (20.04°N, 99.95°E) and Ban Mae Mon (19.85°N, 99.61°E), Meuang district in Chiang Rai Province and Ban Huay Muang (19.14°N, 100.72°E), Tha Wang Pha district, Nan Province. Rodents were killed using isoflurane, following international standards for animal-handling and euthanasia (Sikes, 2016; AVMA Panel on Euthanasia, 2013) and tissues were collected: ears were placed in 70% ethanol and stored at 4 °C and lung, liver and spleen were stored in 70% ethanol at −80 °C (Herbreteau et al., 2011). Free-living chiggers were collected using the black plate method (Gentry, 1965; Uchikawa et al., 1993). Human blood and eschar samples were collected during non-malarial fever studies in Laos (Phetsouvanh et al., 2015) and a Natural Immune Response to Paediatric Scrub typhus study in Thailand and stored at −80 °C (Wangrangsimakul et al., 2020). One chigger sample was collected on the Penghu Islands, Taiwan (23.57°N, 119.64°E) and human samples were also collected from Chiang Rai Province, Northern Thailand, across Laos and one from Green Island, Taiwan (22.66°N, 121.49°E). Chiggers were identified using autofluorescence and bright-field microscopy (Kumlert et al., 2018) with using taxonomic keys (Nadchatram and Dohany, 1974; Vercammen-Grandjean, 1968; Stekolnikov, 2013). Ethical approval was obtained from Kasetsart University Animal Ethics Committee (EC), Bangkok, Thailand for animal collection; the Faculty of Tropical Medicine EC, Mahidol University, Bangkok, the Chiangrai Prachanukroh Hospital EC, the Chiangrai Provincial Public Health EC and the Oxford Tropical Research EC for human samples in Thailand and the Lao National Committee for Health Research for human samples in Laos.

### 2.3 Preparation and quantitation of *O. tsutsugamushi* samples for sequencing

DNA was extracted from individual chiggers, pools of chiggers, rodent tissues and human samples using the Qiagen Blood and Tissue Kit (Qiagen, USA), as follows. Chiggers were rinsed with distilled water and cut through the mid-gut using a sterile 30G needle under a dissecting microscope. Pools were crushed using a sterile polypropylene motorized pestle (Motorized pellet pestle Z35991, Sigma Aldrich, St Louis, MO). Rodent tissues were cut into small pieces (≤10 mg of spleen or ≤ 25 mg of liver or lung). Buffy coat or whole blood was extracted from a starting volume of 200 μL. Eschars were collected either as pieces of crust in 70% ethanol or swabs. Samples were digested with proteinase K for 1 h (whole blood and buffy coat), 3 h (chiggers, rodent tissues and eschar swabs) or overnight (eschar crust), and the rest of the steps followed the manufacturer's protocol. Chigger samples were eluted in 45 μL and other samples were eluted in 100 μL of Qiagen's buffer AE for storage at −20 °C. All samples were analysed using a quantitative real-time PCR targeting the 47 kDa *O. tsutsugamushi* outer-membrane protein (Jiang et al., 2004), on 5 μL (chiggers) or 1 μL of DNA (other samples), using in-house copy-number standards from 10^0^ to 10^6^ in duplicate with two no-template controls included in every run.

*O. tsutsugamushi* (strains UT76 and CRF136) DNA from cultured cells was spiked into chigger DNA in order to create a dilution series with realistic characteristics. DNA was extracted from 20 *O. tsutsugamushi*-PCR-negative chiggers of the genus *Walchia*, pooled and then split into 20 tubes, such that the sample was equivalent to the mean amount of DNA extracted from a chigger. DNA containing 82% *O. tsutsugamushi* sequences as estimated by qPCR and bulk sequencing was used to construct an initial 100,000 copies per μL, which was diluted in chigger DNA to form successive 50,000, 25,000, 10,000, 5000 and 1000 copy per μL solutions.

In the first round of sequencing in this study, the Nextera XT DNA library preparation kit (Illumina Inc., San Diego, USA) methodology was used to prepare libraries, used in the validation of the target enrichment methodology and then in a round of experiments predominantly for human-derived samples. For Nextera XT libraries, DNA was normalized for an input of ≤1 ng in 5 μL across all samples and libraries were prepared following the manufacturer's protocol.

### 2.4 Assessment of target enrichment and sequencing

Library pools for hybridization were first blocked using SureSelect Indexing Blocks #1 and #2 and Integrated DNA Technologies xGen Blocking Oligos, then hybridized for 24 h with the *O. tsutsugamushi* SureSelect panel using the capture module of the SureSelectXT Reagent Kit, HSQ (Agilent). Libraries were washed according to the SureSelect protocol and re-amplified for 14 PCR cycles using Herculase II Fusion DNA Polymerase with Illumina qPCR Library Quantification Primer Premix (KAPA), before sequencing on the Illumina HiSeq4000 platform with paired-end 150 bp reads.

**Fig. 1:**
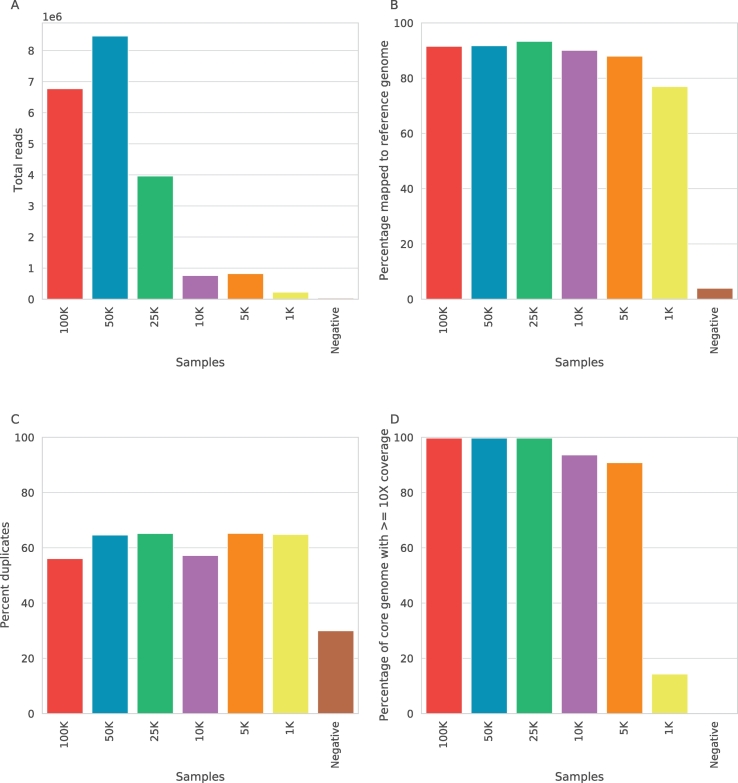
Results from sequencing of spike-in control samples using Nextera library preparation method showing a) total reads produced b) percentage of those reads which mapped to the reference genome c) percentage of the reads which were duplicates and d) the percentage of the core genome covered by 10 or more reads.

After sequencing, raw reads were mapped to the UT76 reference genome (GCF_900327255.1) using BWA MEM v0.7.12 (Li, 2013), then sequencing statistics were calculated using Samtools flagstat v1.8 and GATK v3.7 (McKenna et al., 2010) before and after deduplication using Picard MarkDuplicates v2.0.1. The results of the initial spiked sample sequencing are shown in Fig. 1. Total reads of 2.2 × 10^5^ to 8.5 × 10^6^ were obtained for each sample, with 32–93% of reads mapping to the target genome. Due to the highly repetitive nature of the *O. tsutsugamushi* genome, which varies hugely between strains, we chose to measure coverage statistics by using coverage across 657 core genes previously identified as present in all samples (Batty et al., 2018), covering 685 kb of the 2.2 Mb genome. The proportion of the core genome covered with ≤10 reads ranged from 14.3 to 99.8. The percentage of reads which were identified as sequencing duplicates ranged from 51 to 66%, with a greater duplication rate in the samples with lower quantities of target DNA, as expected.

### 2.5 *O. tsutsugamushi* genomic sequencing in samples from infected chiggers, rodents and humans

The low-input Nextera library preparation method was used next to sequence a set predominantly containing human samples. This generated inconsistent results, thought to be driven by low and inconsistent amounts of input DNA leading to low-complexity libraries, highly variable pooling and ultimately, high sequencing duplication rates. We therefore altered the library preparation to incorporate a whole-genome amplification step followed by a ligation-based library preparation method.

Specimens with input volumes from 40 μL (chiggers) and ~ 50 μL (human samples) to 95 μL (small mammal samples) were vacuum-dried and resuspended in 2.5 μL of TE for WGA using the REPLI-g Single Cell Kit (Qiagen, Hilden, Germany). Amplified samples containing 500 ng mass were fragmented using an Episonic instrument, (EpiGentek, NY, USA) then cleaned using AMPure XP beads (Beckman Coulter, Indianapolis, USA) for library preparation using the NEBNext Ultra DNA Library Prep Kit for Illumina (New England Bioalabs) with a slightly modified protocol using in-house Y-adapters. Libraries were cleaned up and size-selected using AMPure XP beads followed by indexing PCR and cleanup in preparation for pooling and target enrichment.

Validation of the updated method using spike-in samples revealed lower duplication rates (Supplementary Fig. 1 and Supplementary Table 1); therefore all subsequent batches were sequenced with an initial whole-genome amplification step.

**Fig. 2:**
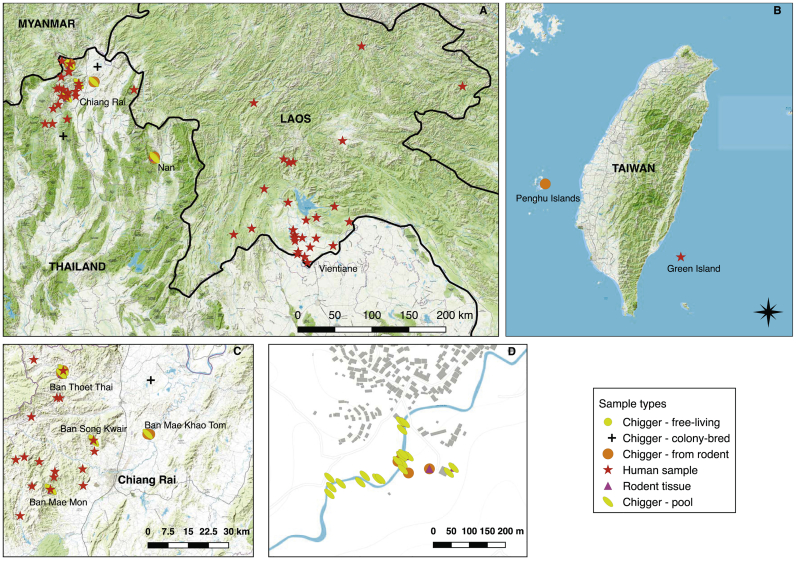
Sample collection locations. A) Southeast Asia with locations in Laos and Northern Thailand, B) Taiwan, C) Chiang Rai Province, with key field sites named, D) Ban Thoet Thai, Chiang Rai Province, site of the greatest number of *O. tsustsugamushi* PCR positive chigger and rodent samples.

A wide selection of samples were used for further testing, including 69 human samples (18 whole blood, 31 buffy coat, and 20 eschar samples) from Thailand, Laos, and Taiwan (Fig. 2 and Supplementary Table 1). Chigger samples came from individual (27) and pooled chiggers (91) collected from animals, infected colony chiggers provided by the Armed Forces Research Institute for Medicine (AFRIMS) in Bangkok, Thailand, and a single free-living chigger. Rodent samples included lung and liver tissues from 7 animals of 3 different species. In some cases we tested multiple samples from the same individual, including paired blood and eschar samples from humans and multiple chigger pools taken from the same animal. All samples were PCR-positive for the 47 kDa gene. The C_t_ values for the samples ranged from 24.6 to 41.3 cycles.

**Fig. 3:**
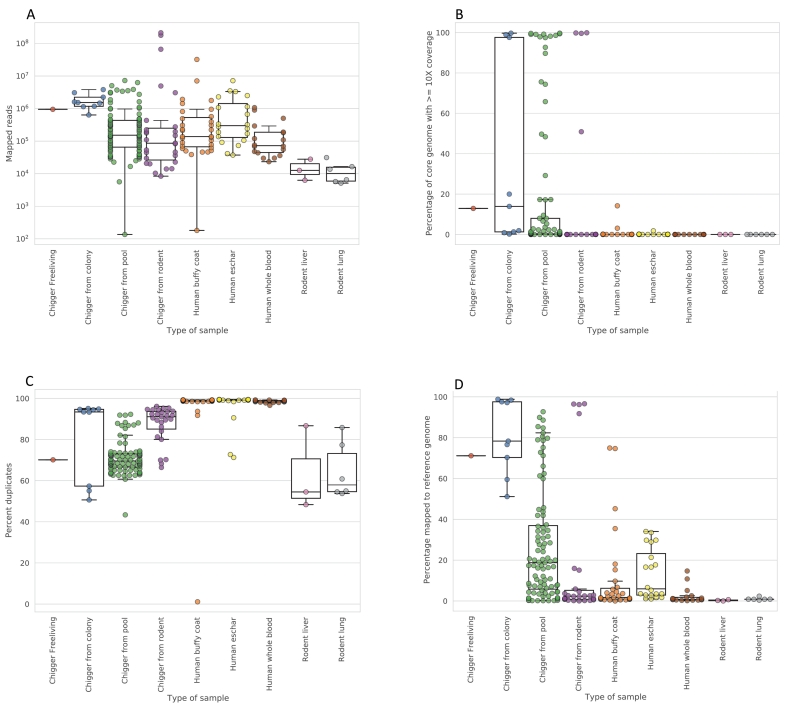
Sequencing statistics for human, chigger, and rodent samples. Panels show a) total number of reads and b) the percentage of reads which were mapped to the reference genome. Panel c) shows the sequence duplication rate and d) shows the coverage of the core genome. Box and whisker plots show the distribution of the data, with the whiskers marking the maximum and minimum values with outliers removed (where outliers are defined as points outside 1.5 interquartile ranges of the lower and upper quartile).

We assessed sequencing outputs based on the number and proportion of reads generated which map to the reference genome, the coverage of the core genes, and the sequence duplication rate (Fig. 3). In most samples, only a small proportion of reads mapped to the reference genome, reflecting the performance of the methodology on samples that in general had very small amounts of *O. tsutsugamushi* sequences. Among the different chigger sample types, colony chiggers performed well, with a high percentage of reads mapped to the reference genome likely reflecting their higher input total copy number and corresponding lower C_t_ (mean 29.4, range 28.6–30.2). Chigger pools and individual chiggers from rodents had high variability but with some samples having high levels of reads mapped to the reference genome and correspondingly a high percentage of the genome covered at 10× coverage. C_t_ values for individual chiggers were higher (mean 36.4, median 37, range 30.2–40.2) compared to chigger pools (mean 31.3, median 30.9, range 24.6–40.3). Among the human samples, buffy coat and eschar samples gave more variable performance, with very few samples having sufficient genome coverage to be used in variant calling, and whole blood performed least well with percentage of the core genome covered at 10× or more under 1% in all samples and median percentage of reads mapped to the reference genome of 0.72%. Rodent tissue samples performed poorly in all cases. The relatively low C_t_ values for colony chiggers and their high core genome coverage may reflect the unusual ecological scenario of long-term colony chiggers that may result in higher loads of *O. tsutsugamushi* than wild chiggers (Supplementary Fig. 2).

We expected a positive association between the rate of reads matching *Orientia* sequences and the number of *Orientia* genome copies detectable by qPCR. We compared the fraction of reads which mapped to the C_t_ values (Supplementary Fig. 3). Colony chiggers had the highest fraction of reads mapped to the reference genome and tended to have the lowest C_t_ (Supplementary Fig. 3). A lower C_t_ (higher input number of genomes) was correlated with the percentage of reads mapped to the reference (Spearman's rank order correlation = −0.70, *p* = 1.05 × 10^−35^) (Supplementary Fig. 3).

The multiple sample types had a wide range of estimated genome copies, as well as different properties such as total DNA content, which change the ratio of target to non-target DNA. Many samples fell near the lower limit of detection of the qPCR assay, with 69/205 (34%) having C_t_ > 35. It appears that a C_t_ of ≥35 results in poor coverage and low percentage mapping to the reference.

### 2.6 Phylogenetic analysis of *O. tsutsugamushi* in diverse samples

Single-nucleotide polymorphisms (SNPs) were identified in the entire set of sequenced samples using Snippy v4.3.6 (Seemann, 2012) and regions falling outside the core genome were masked using Snippy-core. Since many samples had low and fragmentary genome coverage, phylogenetic comparisons were only attempted for a set of 31 samples with >50,000 bases called, comprising 4 chigger pools from Ban Mae Mon, Thailand, 1 human buffy coat sample from Na Meuang, Laos, 1 individual chigger from the Penghu Islands, Taiwan, 4 individual chiggers and 17 chigger pools from Ban Thoet Thai, Thailand, and 4 colony chiggers. The median C_t_ value for these 31 samples was 29.0 (range 25.4–34.2), the distribution of the numbers of core genome positions called appears in Supplementary Fig. 4 and a heatmap of coverage for each core gene is shown in Supplementary Fig. 5. For almost all samples, there is some sequence coverage for each of the core genes, and for those with fewer positions called it is due to incomplete coverage across the genome rather than genes which are completely uncovered in sequencing. A notable exception is sample C0546, which has many genes which have no coverage at all but sufficient coverage in the remaining genes to meet the 50,000 bp threshold. A small number of genes were completely uncovered in multiple samples, most notably several genes which have no coverage in any of the samples taken from the R240 pools from a rodent in Ban Mae Mon. These observations hint at the divergence between samples and the existence of sets of genes whose presence or absence may reflect phylogenetic relationships.

**Fig. 4:**
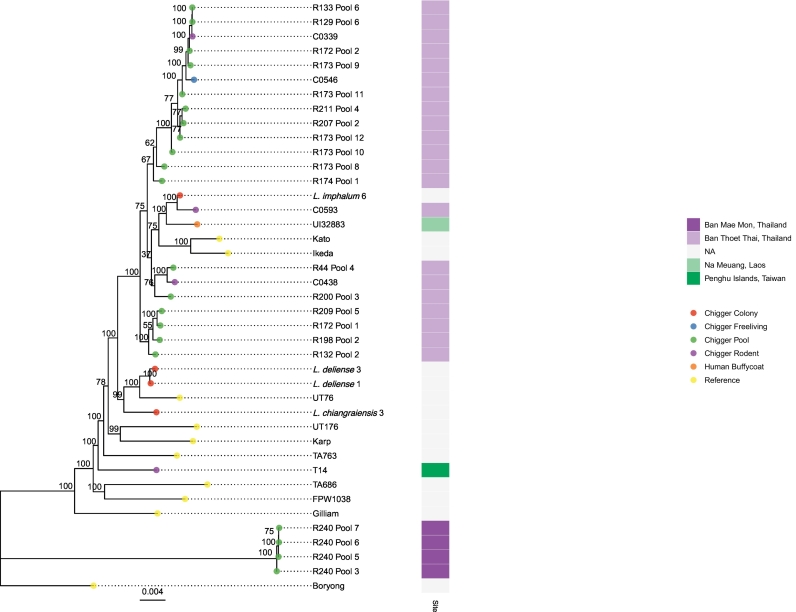
A maximum-likelihood phylogenetic tree produced using IQTREE from all samples that have >50 kb of called positions. The unrooted tree was visualized with the Boryong strain as root. Tip colors represent the source of each sample, and the heatmap shows the site where samples were collected. The node labels show ultrafast bootstrap support values.

A maximum-likelihood (ML) phylogenetic tree of the complete set of called variants in the best-covered 31 genomes was constructed using iqtree v1.3.11 (Nguyen et al., 2015) with the TVM + F + R6 model, selected by ModelFinder Plus (Kalyaanamoorthy et al., 2017) using the log-likelihoods of an initial parsimony tree for many different models and the Akaike information criterion (AIC), corrected AIC and Bayesian information criterion (BIC). The phylogeny is shown in Fig. 4. Branch support values, estimated by an ultrafast bootstrap approximation with 1000 replicates (Hoang et al., 2018), fall below 70% for some branches, indicating some uncertainty in tree topology. The samples include two colony chiggers from the same L. *deliense* colony, which are closely related but not identical (separated by 35 SNPs).

### 2.7 Data availability

The sequences uploaded to generate Agilent SureSelect capture probes are available through Figshare at https://doi.org/10.6084/m9.figshare.12546377. The VCF files and the alignment used to construct the phylogenetic tree are available through Figshare at https://doi.org/10.6084/m9.figshare.13147619. The sequence reads for all strains are available in the Sequence Read Archive under project PRJEB39975. For sequence read sets obtained from human samples, reads mapping to the human genome using Bowtie2 were removed from the data before uploading.

## 3 Discussion

We have successfully developed and tested the first whole-genome sequencing of *O. tsutsugamushi* performed without prior cell culture. The sequence data generated provided an opportunity to compare *O. tsutsugamushi* strains with greater resolution than previously possible.

The sequencing results displayed great variability, with sufficient success to call variants and perform phylogenetic analysis in 31/206 (15%) samples, with a higher proportion of 30/128 (23%) seen for individual and pooled chiggers. The yield of unique on-target reads, particularly at the low copy number dilutions (5000 and 10,000 copies) was higher for WGA before library preparation than for Nextera XT, and the duplication rate was also improved. The low success rate likely reflects very low quantities of *O. tsutsugamushi* DNA present in many samples, especially human samples, and reflects the current limit of our enrichment method, which cannot enrich sufficiently to overcome the low levels of input DNA. While no firm C_t_ cut-off value can be established above which target enrichment sequencing cannot be successfully performed, samples with a C_t_ value of 35 or less are candidates for sequencing. Methods for human and rodent DNA depletion prior to sequence capture may improve the performance of enrichment. The first full genome of L. *deliense* has been published since this array was designed, and this could be used to check for any sequences in the array design which may capture off-target chigger DNA (Dong et al., 2018).

A recent study has reported phylogenetic comparisons of *O. tsutsugamushi* strains from chiggers collected from the same host animal, based on sequencing of a single gene (encoding the 56 kDa antigen) (Takhampunya et al., 2018). Results revealed mixed infections; with some chiggers containing a single genotype and others mixed genotypes. There is also evidence of different *O. tsutsugamushi* 56 kDa type-specific antigen genotypes being maintained and transmitted transovarially in colony chiggers (Takhampunya et al., 2016).

The sequence capture probes used in this experiment were designed when only two complete genomes were available to use in the design process. Of the incomplete assemblies included in the design process, two strains have been removed from RefSeq due to problems with the assembly, and more complete genomes are now available. These genomes were completed using PacBio long-read sequencing technology, which can resolve the complete genome structure including the many repeat regions, but this method can currently be used only on samples which have been cultured. A new probe design using the same approach but more genomes may improve the capture efficiency. We chose to design probes to hybridise to all known *O. tsutsugamushi* genomes to capture the most complete genomes possible, however for many experiments a smaller number of genes would be sufficient to give phylogenetically informative results. Using the extra genomes now available, a core gene multilocus sequence typing (cgMLST (de Been et al., 2015)) scheme could be developed to identify the optimal genes for a typing scheme and to give a standardized system to compare strains globally. This would also allow for the use of a smaller sequence capture probe set, lowering the cost per genome.

Despite the poor performance for the target enrichment sequencing on some samples, we were able to generate a phylogeny using 30 chigger samples, 1 human sample, and 8 complete reference genomes, which represents the first genome-scale phylogenetic analysis of *O. tsutsugamushi* from chiggers. Among the 31 best-sequenced samples, >98.5% of the core genes of the reference sequence were covered by at least one read at all positions. For most samples, the regions of no coverage were confined to a very few genes, some of which were present in all samples. Intriguingly, for chigger pools from Ban Mae Mon (R240), more genes were incompletely covered, and most of these were present in all samples, even though the total volume of on-target reads (equivalently, the average coverage of the core genome) was similar in these samples as in other high-performing samples. This could be due to diversity in these genes beyond the limits that our probes could capture; however, the sequence capture probes have been shown to be effective at up to 20% sequence divergence (Bonsall et al., 2015), and the overall diversity between our phylogenetic samples is well below this limit. It is more likely that the set of core genes determined from the known complete genomes is not universally present in all strains.

The study included strains sequenced from chiggers collected from a single host animal, strains from chiggers from several animals at a single study site of <10km^2^ and from two sites 45 km apart. Samples from Ban Mae Mon are clearly distinct from samples from Ban Thoet Thai, which group together (Fig. 4). All the chigger pools and individuals from Ban Thoet Thai consisted of the known vector L. *imphalum* (with or without some *Walchia* species). The Taiwanese chigger was the known human vector L. *deliense*. The R240 pools from Ban Mae Mon, which form a distinct cluster separate from all other samples, were collected from the scansorial tree shrew *Tupaia glis* and consisted of L. *turdicola* and *Helenicula naresuani* chiggers – neither known to be human vectors nor previously reported as being infected with *O. tsutsugamushi*. The reference genomes, which were collected from five different countries between 1943 and 2010, are spread throughout the tree and many are more closely related to the samples from Ban Thoet Thai than the samples from Ban Mae Mon are to those samples. A possible explanation for this is that *O. tsutsugamushi* has been previously introduced into these two locations from divergent sources and continues to evolve locally on a small scale, and larger-scale *O. tsutsugamushi* movement between locations is a rare event due to the restricted range of the host species.

Important questions remain about the role of recombination between strains in infected chiggers and to what extent the accessory genome of *Orientia* is open or closed. The sequence capture approach used in this study does not recover the complete accessory genome, and hence cannot assist with the latter question. The accumulation of more high-quality sequences may allow characterization of the recombination landscape. However, *O. tsutsugamushi* genomes are known to have poorly conserved synteny, which is likely to complicate analysis of incomplete genomes.

Among captured sequences, pairwise divergences were in the range of 0–4%, well within the reach of probe-based sequence enrichment for pathogen genomics (Bonsall et al., 2015). This illustrates the robustness and adaptability of probe-based sequence enrichment, providing a means for genome-wide amplification of sequence information without the need to validate a very large number of PCR primers, any of which could fail because of hitherto-uncharacterised sequence variation.

The methods developed in this project have, for the first time in scrub typhus research, demonstrated phylogeographic clustering of *O. tsutsugamushi* strains at international, provincial and highly local scales. This shows that both closely related and more distantly related strains may co-exist in one site. As methods improve and can be applied to a greater range of samples, particularly sympatric rodents and exposed humans, further insights into this fascinating phylogeographic variation will be revealed with important consequences for diagnostic tests and vaccine development strategies.

The following are the supplementary data related to this article.Supplementary Table 1 Sequencing metadata for all samples, including sample type 6 and location of collection. Supplementary Table 1

Supplementary material Image 1

## Financial support

This study was supported by Ivo Elliott's Wellcome Trust Research Training Fellowship (105731/Z/14/Z and in part by Core Awards to the Wellcome Centre for Human Genetics (090532/Z/09/Z and 203141/Z/16/Z) and by the Wellcome Trust Core Award Grant Number 203141/Z/16/Z with additional support from the NIHR Oxford BRC. The views expressed are those of the author(s) and not necessarily those of the NHS, the NIHR or the Department of Health.

## Conflict of interests

IE, NT, MdC, PL, DHP, NDJP, PNN, RB, EMB - none.
